# Pre-clinical validation of B cell maturation antigen (BCMA) as a target for T cell immunotherapy of multiple myeloma

**DOI:** 10.18632/oncotarget.25359

**Published:** 2018-05-25

**Authors:** De-Xiu Bu, Reshma Singh, Eugene E. Choi, Marco Ruella, Selene Nunez-Cruz, Keith G. Mansfield, Paul Bennett, Nathanial Barton, Qilong Wu, Jiquan Zhang, Yongqiang Wang, Lai Wei, Shawn Cogan, Tucker Ezell, Shree Joshi, Kellie J. Latimer, Brian Granda, William R. Tschantz, Regina M. Young, Heather A. Huet, Celeste J. Richardson, Michael C. Milone

**Affiliations:** ^1^ Novartis Institutes for Biomedical Research, Cambridge, MA 02139, USA; ^2^ China Novartis Institutes for Biomedical Research, Shanghai 201203, China; ^3^ Center for Cellular Immunotherapies, Perelman School of Medicine at the University of Pennsylvania, Philadelphia, PA 19104, USA; ^4^ Department of Pathology and Laboratory Medicine, Perelman School of Medicine at the University of Pennsylvania, Philadelphia, PA 19104, USA

**Keywords:** BCMA, T cell, CAR, multiple myeloma

## Abstract

Multiple myeloma has a continued need for more effective and durable therapies. B cell maturation antigen (BCMA), a plasma cell surface antigen and member of the tumor necrosis factor (TNF) receptor superfamily, is an attractive target for immunotherapy of multiple myeloma due to its high prevalence on malignant plasma cells. The current work details the pre-clinical evaluation of BCMA expression and development of a chimeric antigen receptor (CAR) targeting this antigen using a fully human single chain variable fragment (scFv). We demonstrate that BCMA is prevalently, but variably expressed by all MM with expression on 25–100% of malignant plasma cells. Extensive Immunohistochemical analysis of normal tissue expression using commercially available polyclonal antibodies demonstrated expression within B-lineage cells across a number of tissues as expected. Based upon the highly restricted expression of BCMA within normal tissues, we generated a set of novel, fully human scFv binding domains to BCMA by screening a naïve B-cell derived phage display library. Using a series of *in vitro* and pre-clinical *in vivo* studies, we identified a scFv with high specificity for BCMA and robust anti-myeloma activity when used as the binding domain of a second-generation CAR bearing a CD137 costimulatory domain. This BCMA-specific CAR is currently being evaluated in a Phase 1b clinical study in relapsed and refractory MM patients (NCT02546167).

## INTRODUCTION

Multiple myeloma (MM) is the second most common hematological malignancy in the US with about 22,000 new diagnoses per year. MM derives from the clonal expansion of malignant bone marrow plasma cells (PC), and is associated with clinical complications including hypercalcemia, renal insufficiency, symptomatic anemia, destructive lytic bone lesions, and increased susceptibility to infection. Autologous stem-cell transplant (ASCT) and the introduction of novel agents (proteasome inhibitors, immunomodulatory drugs, and monoclonal antibodies such as daratumumab or elotuzumab) have markedly delayed the natural progression of this disease [1]; However, MM remains largely incurable with the majority of patients relapsing after multiple lines of therapy and eventually succumbing to progressive disease. Thus, the development of novel therapeutics specifically targeting multiple myeloma is warranted.

Chimeric antigen receptor (CAR) T cells are a novel immunotherapy approach that involves redirecting patient T cells to recognize and kill cancer cells. The CAR is a synthetic protein that includes an antigen-recognition domain, typically formatted as a single chain variable fragment (scFv), and a signaling domain that drives the activation of the T cells (CD3z and costimulatory domains such as 4-1BB and CD28). Upon CAR-mediated recognition of the neoplastic cells, CAR T cells become activated and exert their effector functions to kill tumor cells, proliferate and establish long-term memory [2]. In the last few years, anti-CD19 CART therapies (e.g. CTL019, KTE-C19) have shown impressive clinical results in the setting of B cell malignancies, in particular B-ALL, with multiple groups showing high rates of complete response with deep molecular remission in most of the patients [3–7]. Expanding this type of therapy to MM is of high interest.

BCMA, a member of the tumor necrosis factor (TNF) receptor superfamily, is an attractive target for CART mediated therapy in MM. BCMA is expressed in MM cells from most patients as revealed through both RNA and protein measures [8–12] and is found at elevated levels in sera of patients [13]. Anti-BCMA antibodies have been found in patients who achieved complete remissions after allogeneic transplant through a graft-versus-MM response [8]. The BCMA ligands APRIL and B-cell activating factor (BAFF) are produced by cells in the tumor environment of the bone marrow and occasionally by MM cells themselves [14]. APRIL and BAFF protect MM cells from apoptosis [10]. Following stimulation with APRIL or BAFF, BCMA becomes a trimer, eliciting a signaling cascade involved in the activation of MAP kinases and the induction of anti-apoptotic proteins, such as Bcl-2 and Bcl-XL [15]. BCMA was also identified by chromatin immunoprecipitation (ChIP) analysis to be a target of IRF4, a transcription factor that is required for MM survival [16]. Finally, anti-tumor activity has been achieved with BCMA-directed or ligand-directed therapeutic agents in both pre-clinical MM models as well as in early phase human clinical trials that include CAR-T cell therapy [17–22].

BCMA is expressed by B-lineage derived cells [17] with expression limited to plasmablasts and plasma cells, along with a subset of memory B cells [23, 24]. BCMA expression can be induced by stimulation with cytokines and is important for the survival of long-lived plasma cells in the bone marrow [25]. BCMA expression on tumor-associated endothelial cells [26], keratinocytes [27] and adipocytes [28] has also been reported in the literature, making thorough evaluation of the tissue distribution imperative for translation of therapies targeting this molecule into the clinical setting.

Based on the apparent role of BCMA in MM pathogenesis, and the limited reported expression on normal tissues, we set out to validate the expression of BCMA in MM and normal tissues followed by generation of a highly potent, and selective BCMA-targeting CAR for adoptive T-cell therapy in myeloma. Previous clinical stage CAR constructs were derived from the conversion of published monoclonal antibody sequences into scFv format binding domains. We report the preclinical evaluation of a CAR bearing a novel, fully human scFv that is currently undergoing clinical evaluation in a Phase Ib study in relapsed and refractory MM patients (NCT02546167).

## RESULTS

### BCMA expression is prevalent, but variable across malignant plasma cells in MM

**Figure 1:**
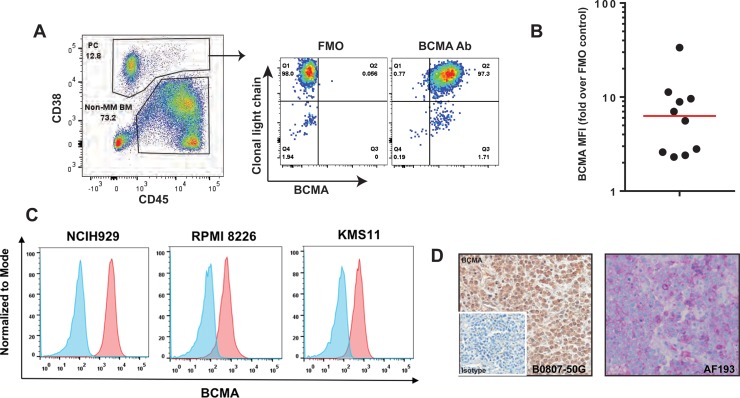
BCMA expression on MM patient samples (**A**) An example of flow cytometry analysis of a bone marrow (BM) sample from an MM patient is shown. Plasma cells (PC; both malignant and non-malignant) were identified by gating bone marrow on live single cells followed by using CD38 and CD45 staining as shown. Malignant PCs were evaluated for BCMA expression with fluorescence minus one, FMO, used as control. (**B**) Ten consecutive MM patients were analyzed for BCMA expression as in Figure 1A. BCMA median expression was 86% (red line). (**C**) BCMA expression (dark histogram) on 3 established multiple myeloma cell lines assessed by flow cytometer. The lighter histogram shows staining of a matched isotype control. (**D**) Representative immunohistochemical staining of a bone marrow biopsy specimen stained for BCMA using two commercially available antibodies, B0807-50G (brown staining, primaryantibody concentration of 0.6 μg/ml, US Biological) and AF193 (magenta staining, primary antibody concentration of 2.0 μgml, R&D Systems).

To evaluate the expression of BCMA in MM, we analyzed bone marrow (BM) mononuclear cells obtained from individuals with MM by flow cytometry. Normal and malignant plasma cells (PC) were identified using a sequential gating strategy. As shown in Figure 1A, BCMA is highly expressed on normal and malignant PCs with the latter being defined by clonally restricted light chain expression. Analysis of BM aspirates from 10 individuals with MM (see Supplementary Table 1) revealed the high prevalence of BCMA expression across different tumors (Figure 1B). However, detectable expression varied from 25% to 100% of malignant PCs. Evaluation of the established MM cell lines, NCI-H929, RPMI-8226 and KMS11 demonstrated uniform BCMA expression that was confirmed by RT-PCR (Figure 1C and data not shown). Immunohistochemistry using commercially available antibodies to BCMA that were distinct form that used for flow cytometry confirmed the expression of BCMA on malignant PCs in BM (Figure 1D). BCMA was not detectable on non-malignant bone marrow stem or progenitor cells from healthy donors (data not shown).

### BCMA expression is highly restricted to B-lineage cells in normal tissues

**Figure 2:**
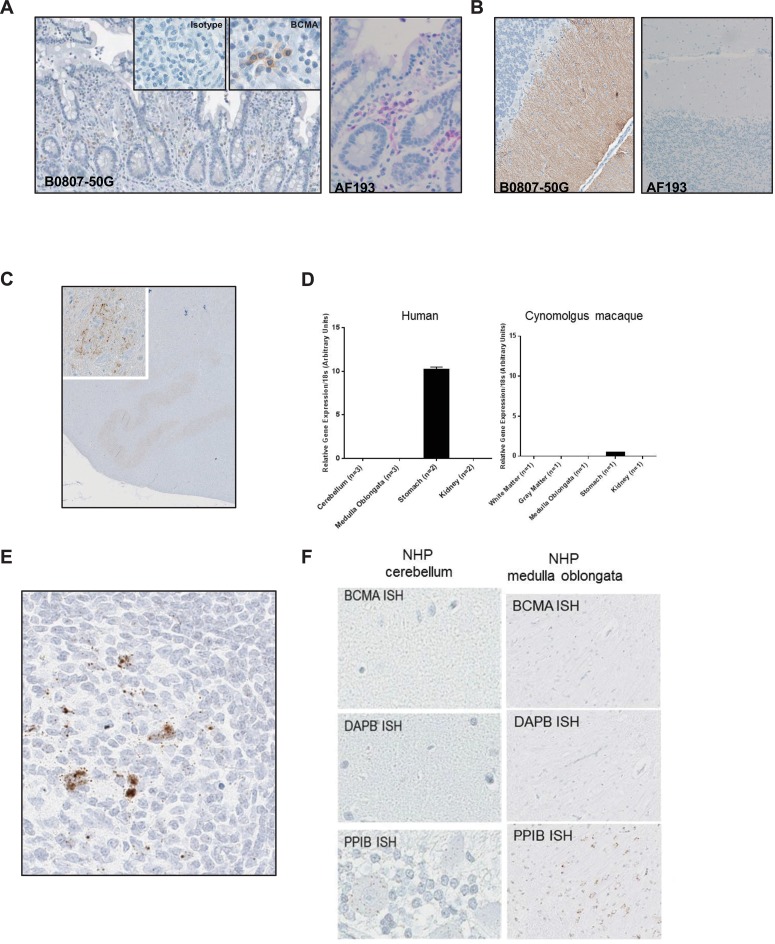
Immunohistochemical staining with two commercially available anti-BCMA antibodies show disparate staining within the brain (**A**) IHC staining of small intestine showing plasma cells using B0807-50G (brown staining) and AF193 (magenta staining). (**B**) IHC staining of cerebellum. (**C**) IHC staining of the NHP (*M. fascicularis*) midbrain using B0807-50G showing reactivity within neuronal cell bodies within the inferior olivary nucleus (ION). (**D**) Expression of BCMA mRNA within indicated tissues measured by quantitative PCR. Assay was performed in triplicate (mean ± standard deviation). Results are expressed relative to 18S ribosomal RNA expression. (**E**) ISH for BCMA mRNA using a target specific probe (Advanced Cell Diagnostics) on normal NHP small intestine. (**F**) ISH for BCMA mRNA on NHP cerebellum and medulla oblongata. Probes specific for the bacterial protein, DAPB and the cyclophilin, PPIB were used as negative and positive controls, respectively, for RNA quality.

To further examine BCMA normal tissue distribution, two commercially sourced anti-BCMA polyclonal antibodies (AF193 and B0807-50G) as shown in Figure 2A, were used to examine a wide range of tissues by immunohistochemical analysis using normal human tissue microarrays (TMA). Immunoreactivity was observed across several normal tissues as shown in Table 1 with reactivity restricted to normal plasma cells. No expression was detectable in endothelial cells, keratinocytes or adipocytes within any of the tissues despite previous reports [26–28]. However, polyclonal antibody B0807-50G demonstrated immunoreactivity on Brunner's and salivary glands and within the cerebellum associated with the climbing fibers. More detailed analysis of central nervous system tissue using human and non-human primate tissue confirmed the reactivity within the cerebellar climbing fibers as well as focal reactivity within the neurons of the inferior olivary nucleus (ION) as shown in Figure 2B and 2C. Given this unexpected immunoreactivity with this antibody, we performed a more detailed analysis of BCMA expression by both quantitative PCR and RNA *in situ* hybridization. We were unable to confirm the expression of BCMA mRNA in glandular tissue nor within any brain tissue as illustrated in Figure 2D–2F. We attempted to identify the binding targets of this commercial antibody within the ION and cerebellum by immunoprecipitation from tissue homogenates followed by mass spectrometry; however, these studies were inconclusive (data not shown). Based upon these aggregate studies, we concluded that BCMA is a highly restricted target with normal tissue expression limited to normal B cells and plasma cells. The immunoreactivity observed using the commercial antibody B0807-50G in the cerebellum and ION most likely represents binding to a cross-reactive epitope rather than BCMA.

**Table 1:** Tissue Microarray Analysis of Normal Tissues using chimeric rabbit antibody bearing BCMA10 scFv

Tissue	Staining	Pattern
adipose	Negative	
adrenal	Negative	
aorta	Negative	
cerebellum	Negative	
cerebral cortex	Negative	
esophagus	Negative	
heart-left ventricle	Negative	
heart atrium	Negative	
kidney	Negative	
liver	Negative	
lung	Positive	Rare positive cells within the bronchial-associated lymphoid tissue
lymph node	Positive	Rare positive cells
ovary	Negative	
salivary gland	Negative	
skeletal muscle	Negative	
skin	Negative	
spinal cord	Negative	
spleen	Positive	Rare positive cells
stomach	Positive	Scattered positive cells within the mucosa-associated lymphoid tissue
thymus	Negative	
thyroid	Negative	
tongue	Negative	
uterus	Negative	
pancreas	Negative	
small intestine	Negative	
eye-retina	Negative	

### Discovery of novel human BCMA-binding domains

**Figure 3:**
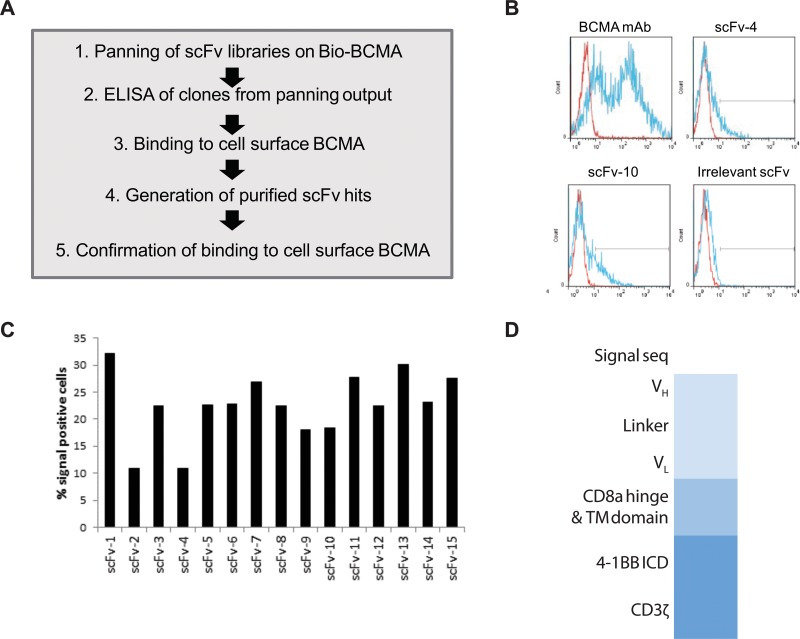
Identification of ScFv clones from human B cell antibody libraries that bind to cell surface-expressed BCMA (**A**) Flow chart of steps taken in the library panning and screening process. (**B**) Histograms of two representative purified scFv-His proteins (clones scFv-4 and scFv-10) analyzed by flow cytometry for binding to BCMA that is transiently expressed on HEK293E cells (blue) or untransfected HEK293E cells (red). Transient expression of BCMA on cells was confirmed using a mouse anti-BCMA mAb (clone 19F2, Biolegend) as a positive control. An irrelevant scFv-His protein was used as a negative control against which the positive gate was set (black line in histograms). (**C**) Summary of the percentage of BCMA-expressing cells (% positive) detected by each of the 15 purified scFv clones analyzed as in panel b. (**D**) Schematic of the CAR construct design. The scFv sequences identified in Figure 2 were cloned in the VH to VL orientation upstream of the CD8a hinge and transmembrane domains (TM), followed by the 4-1BB and CD3 zeta intracellular signaling domains.

Based upon the highly restricted normal tissue expression of BCMA along with its frequent, high expression on malignant plasma cells, we undertook an effort to generate a CAR targeting BCMA for adoptive T cell therapy of multiple myeloma. To minimize the clinical risk of CAR directed immunity that could result in anaphylaxis [29] or immune-mediated loss of CAR T cells [30], we screened a library of fully human immunoglobulin sequences to obtain novel BCMA-binding single chain variable fragments (scFv). The human B-cell derived scFv library was screened using phage display technology [31] as outlined in Figure 3A. Phage clones that bound to recombinant BCMA protein by ELISA were sequenced and found to comprise 135 unique sequences. These phagemid clones were then interrogated for their binding to BCMA that was transiently expressed on HEK293E cells as periplasm protein preparations (data not shown). 15 unique clones that bound to BCMA-producing cells were subsequently reformatted for expression and purification as His-tagged scFv in E.coli, and then re-tested for binding to BCMA transiently expressed on HEK293E cell (Figure 3B). Although binding as assessed by flow cytometry is weak due to the monovalent nature of the scFV, all 15 scFv-His proteins were confirmed to bind to BCMA-transfected HEK293E cells (Figure 3C), but not to parental HEK293E cells (data not shown). CAR constructs were engineered from the 15 BCMA-binding scFv clones by gene synthesis and cloned into a previously described lentiviral expression vector [32]. The resultant CAR constructs contain an extracellular hinge and transmembrane region derived from the CD8 receptor linked to an intracellular bipartite signaling chain of CD3-ζ and 4-1BB intracellular signaling domains as shown schematically in Figure 3D. This intracellular signaling cassette was selected based on the durable clinical responses observed with CD19-directed CAR constructs containing these domains [33].

### Assessment of activity of BCMA-targeting CART cells

**Figure 4:**
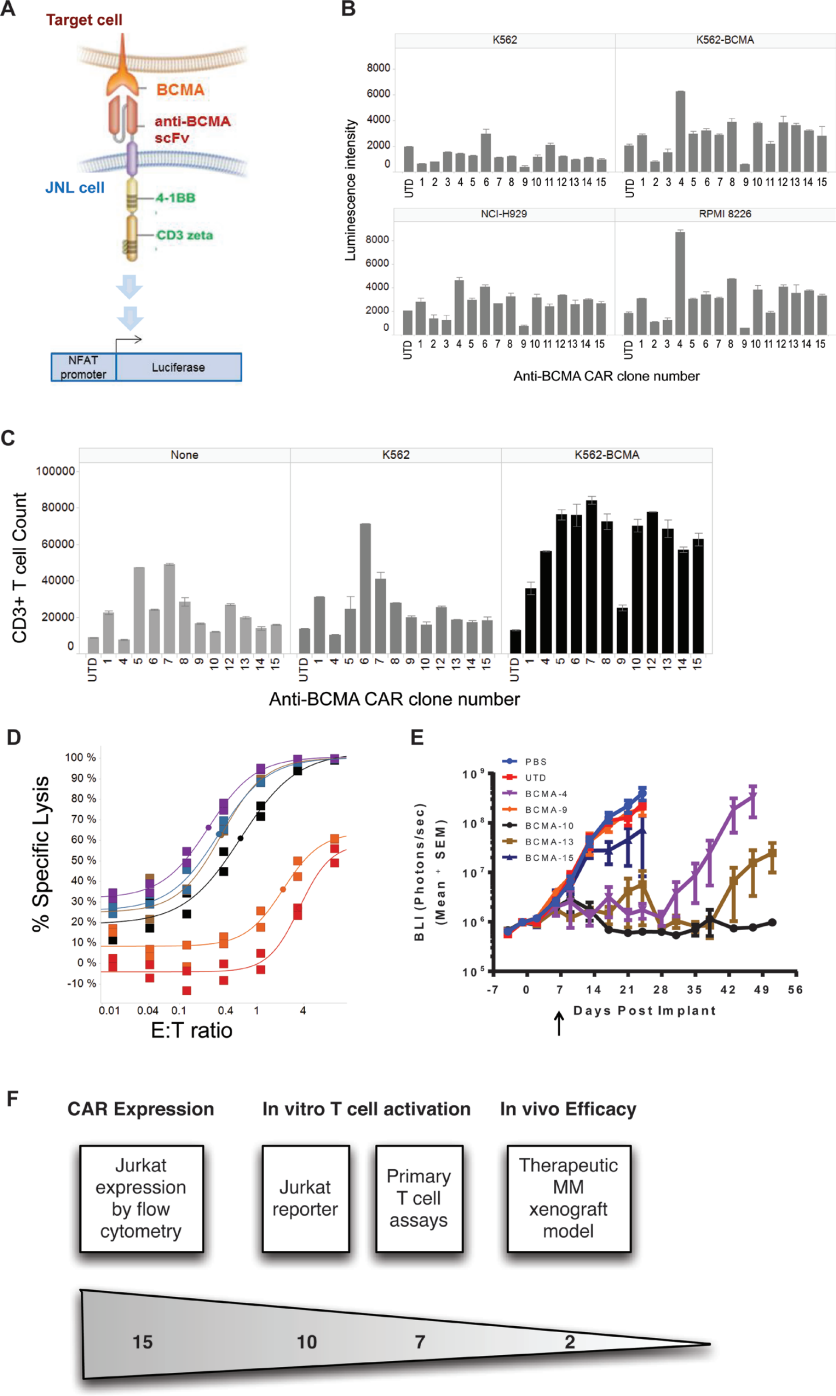
Identification of active and inactive clones using a reporter assay system (**A**) Schematic of the reporter assay. Jurkat cells containing the luciferase gene driven by the signaling-responsive NFAT promoter (termed JNL cells), were transduced with the various CAR constructs. Binding between the CAR construct and its cognate cellular antigen (BCMA on target cells) leads to luciferase expression in the JNL cells. (**B**) CAR clones were evaluated in the JNL reporter assay for antigen-dependent activity. JNL cells containing the indicated CAR clones with expression levels shown in C, or untransduced JNL cells (UTD) were co-cultured with target cells lines (K562, K562-BCMA, NCI-H929, or RPMI8226) and luciferase activity was measured as luminescence intensity. Clones were considered active when the luminescence intensity exceeded 1.25-fold the level of UTD cells in the presence of antigen-expressing cells. Clones were considered non-specific when the luminescence intensity exceeded 1.25 fold the level of UTD cells in the presence of antigen negative K562 cells. (**C**) T cells expressing the indicated CAR clones were evaluated for their ability to proliferate without antigen when co-cultured with no target cells (none), antigen negative irradiated cells (K562). Irradiated antigen-expressing cells (K562-BCMA) were used as a positive control. Proliferation was determined by counting CD3+ cells relative to CountBright Absolute Counting Beads. Untransduced T cells (UTD) revealed the basal level of CAR-independent effects of each target cell line on T-cell proliferation. (**D**) The ability of T cells expressing the indicated CAR clones to mediate cell lysis was evaluated against the KMS-11 target cell line expressing fire fly luciferase (KMS-11-luc). CART cells were co-cultured with KMS-11-luc target cells at the indicated E:T ratios, and % cell killing, determined by the difference in luciferase signal between target cells without effector T cells (control) and with effector T cells (experimental) expressed as a percent of the control, was measured as a surrogate for target cell lysis. UTD represents untransduced T cells. Individual data points represent the mean with the curve fit using an Emax model. (**E**) KMS11-luc cells were implanted in NSG mice and body luminescence (BLI) was monitored over time as a measure of tumor burden. CART cells (1.5 × 10^6^ CAR+ cells in 5 × 10^6^ total T cells), untransduced T cells (UTD; 5 × 10^6^ cells) or PBS were dosed intravenously on day 7 after implantation of 1 × 10^6^ tumor cells. 7 of mice were enrolled in each arm of the study. All data are expressed as mean ± standard error of the mean (SEM). This experiment is representative of 2 independent experiments.

As a first pass evaluation of the selected scFv clones expressed in the CAR format, we used a Jurkat cell reporter system (JNL) that has stable expression of gene cassette comprised of a nuclear factor of activated T cells (NFAT) responsive promoter that drives expression of a luciferase reporter gene (Figure 4A). CAR expression in transduced JNL cells was confirmed for all of the selected clones except clone 3 with 29–70% of cells showing detectable expression across the different clones (Supplementary Figure 1). These transduced JNL cells were co-cultured with BCMA-expressing cells (K562-BCMA, NCI-H929 and RPMI-8226) or control cells lacking BCMA expression (K562). Untransduced JNL cells were used to assess background activity. A clone was considered active if the average activity of duplicates exceeded 1.5-fold the average activity of duplicates derived from untransduced cells in response to any antigen-positive cell lines tested. Clones 1, 4, 5, 6, 7, 8, 10, 12, 13, 14, and 15 were active in this assay while clones 2, 3, 9, and 11 were inactive (Figure 4B). The level of activation seen was not solely attributable to level of CAR expression nor to the level of BCMA expression on target cells.

The selectivity of activation of T cells by the CAR constructs was determined by assessing the ability of the primary human T cells transduced with the different CAR clones to proliferate when exposed to BCMA-expressing cells but not to antigen negative target cells (Figure 4C). A significant level of BCMA target-independent proliferation (>2.5 fold the proliferation level of untransduced cells) was seen for T cells containing CAR clones 1, 5, 6, 7, 8, and 12 in either the absence of target cells (none) or in the presence of antigen-negative K562 cells. Although the mechanisms for this observed independence from BCMA target for proliferation is unknown, similar antigen-independent effects on proliferation and signaling with scFv-based CARs have been reported [34, 35]. Spontaneous scFv aggregation or weak binding to antigens that were not detected in earlier screening assays represent two possible mechanisms for the observed activation. Regardless of mechanism, these clones were eliminated from further consideration. Clone 9 showed minimal proliferation in this assay consistent with the minimal activation within the JNL assay. Clones 4, 10, 13, 14, and 15 showed antigen-specific proliferation of greater than 2.5 fold relative to untransduced control cells. These clones were therefore selected for further evaluation of effector function using both *in vitro* and *in vivo* assays.

Primary human T cells expressing the CARs incorporating scFv clones 4, 10, 13, or 15 all show specific cytolytic activity against the KMS-11 cell line that is above the level observed with untransduced T cells (UTD) or scFv clone 9 CAR, which also lacked activity in both the JNL and proliferation assays (Figure 4D). Similarly, cytokine production in response to BCMA was high in the selected clones 4, 10, 13 and 15 compared with little to no antigen-induced cytokine for UTD or clone 9 CAR cells (Supplementary Figure 2). In order to test the durable cytolytic activity of the selected BCMA-targeting CAR constructs, clones 4, 10, 13, and 15 were further tested *in vivo* for anti-tumor activity against a disseminated KMS-11-luc multiple myeloma xenograft model. The luciferase reporter allows for monitoring of disease burden by quantitative bioluminescence imaging (BLI), which demonstrates predominately bone marrow and splenic disease in this model. Several clones showed potent activity in this model with clone 10-derived CART cells showing the most durable anti-tumor activity (Figure 4E). Monitoring for *in vivo* expansion and persistence of CART cells demonstrated a correlation with the anti-tumor activity with clone 10 showing the greatest CD8+ T cell persistence (Supplementary Figure 3). The enhanced persistence of CD8+ T cells compared with CD4+ cells was consistent with observations in other models where 4-1BB signaling has been implicated in CD8+ T cell memory formation [36, 37]. This durable anti-tumor activity for clone 10 was confirmed in a second experiment using an independent donor (data not shown). Based on this robust *in vitro* and *in vivo* activity, clone 10 was selected for more detailed characterization.

### Further Characterization of the anti-BCMA CAR Clone 10 (CAR-BCMA10)

**Figure 5:**
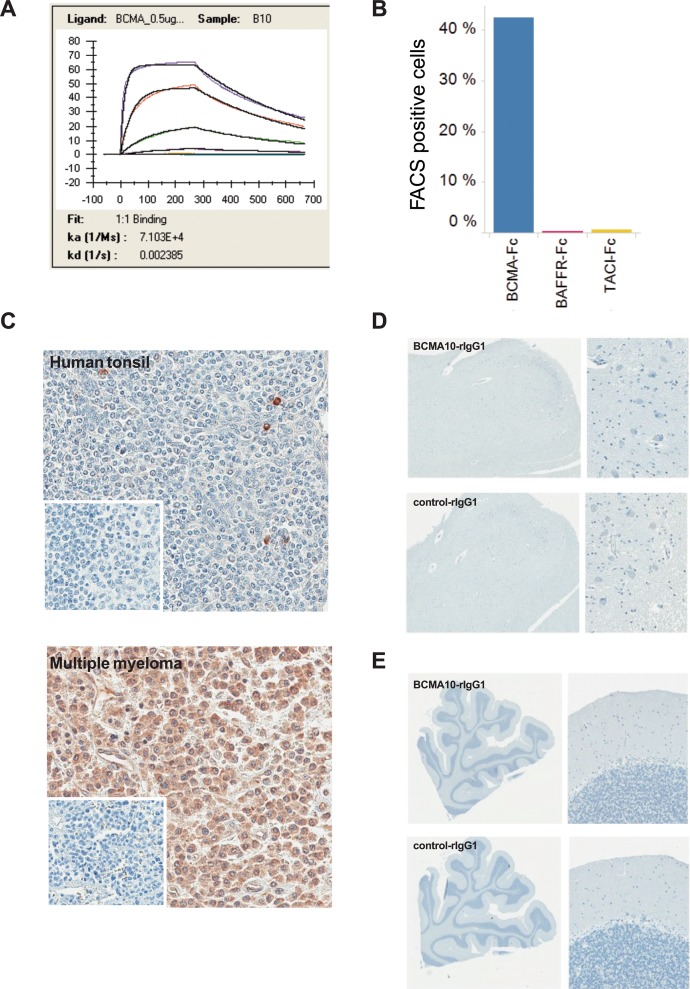
Evaluation of binding specificity and affinity of the clone 10 scFv (**A**) Biacore T200 SPR sensogram for the interaction between clone 10 scFv and recombinant human BCMA. A purified recombinant His-tagged protein containing the scFv of clone 10 was used to determine the binding affinity to recombinant BCMA protein. hBCMA-Fc was immobilized on the chip surface via biotin:streptavidin interaction and clone 10 scFv was flowed over the chip at 1:3 dilutions. Shown are the association constant (ka) and disassociation constant (kd) determined after fitting to a 1:1 binding model used to determine the apparent affinity to rhBCMA-Fc. (**B**) CAR clone 10 was transduced into Jurkat cells and incubated with recombinant Fc-tagged BCMA, BAFFR, or TACI to assess the binding of these proteins to the cell surface-expressed CAR. Binding was detected using an anti-Fc antibody by flow cytometry. The percent of cells with a fluorescence level above the untransduced Jurkat cells is shown. (**C**) IHC staining of human tissue with the clone 10 chimeric antibody demonstrates scattered positive cells in the normal human tonsil (grade 1) and intense uniform staining of multiple myeloma tissue (grade 5) (insert: irrelevant antibody negative control). (**D**) IHC staining of normal human medulla oblongata with a chimeric antibody containing the clone 10 scFv. (**E**) IHC staining of normal cerebellum with a chimeric antibody contain the clone 10 scFv.

Clone 10 scFv binds to recombinant BCMA protein with an affinity of 33 nM (Figure 5A). When expressed as a CAR, clone 10 scFv shows selective binding to BCMA compared with the closely related TNF receptor family members, BAFFR and TACI (Figure 5B). As BCMA can be shed from the cell surface as soluble BCMA (sBCMA) in patients with MM, we evaluated the effect of sBCMA on Clone 10 CAR function *in vitro*. No significant effect of sBCMA on cytotoxic function or cytokine stimulation by BCMA+ cells could be detected with sBCMA concentrations up to 500 ng/mL (Figure 4F, Supplementary Figure 4). The ability of the BAFFR and TACI proteins to bind to antibodies specific for these proteins was confirmed by ELISA (data not shown) confirming the presence of protein that is likely properly folded. To examine the distribution of clone 10 binding across normal human and cynomolgus macaque tissues by immunohistochemistry (IHC), Clone 10 scFv was converted into a chimeric rabbit monoclonal antibody by fusing the scFv sequence with a rabbit IgG1 Fc region. Validation of this chimeric antibody for specificity of staining was performed on cell lines (Supplementary Figure 5) and on human tissue sections, where reactivity was seen on scattered cells in normal tonsil and abundantly on malignant plasma cells in multiple myeloma (Figure 5C). No reactivity was seen in the ION (Figure 5D) or cerebellum (Figure 5E). Staining of a human tissue microarray produced reactivity on plasma cells in spleen, lymph node, lung and gut associated lymphoid tissue (Table 1). An irrelevant human scFV rabbit chimeric control antibody was used in parallel IHC studies, and this control antibody produced no appreciable staining across the same tissues analyzed (Figure 5C–5E and data not shown). To further assess potential off-target binding, the clone 10 scFv was assessed using a plasma membrane protein array assay (Retrogenix, Whaley Bridge, UK) containing more than 4,500 full length proteins individually over-expressed in human cells. BCMA was identified with moderate binding, but no other significant interactions were identified (data not shown).

## DISCUSSION

Therapeutic options in multiple myeloma are rapidly expanding with the development of targeted agents that exploit antigens on the surface of myeloma cells. Elotuzumab, a monoclonal antibody targeting the CS1 antigen on MM cells, showed a 30% reduction in risk of disease progression or death when combined with lenalidomide and dexamethasone [38]. Daratumumab, which targets the MM antigen CD38, showed significant activity as a single agent in patients with refractory disease [39, 40]. Both drugs recently received FDA approval in the US. Despite these advances, there remains a need for therapies with the potential to accomplish more durable responses, as nearly all MM patients eventually succumb to progressive disease. The potential of adoptive cell therapy, which has demonstrated greater durability of response in B-cell malignancies than monoclonal antibody therapies [41], has been explored clinically in MM. Noonan *et al*. observed clinical efficacy using an approach whereby lymphocytes (presumably enriched for tumor-reactive T cells) were isolated from patient marrow, *ex vivo* activated, and adoptively transferred [42]. However, the benefit of such an approach over ASCT remains to be demonstrated with further clinical testing. T cells engineered to express a high affinity TCR that recognizes the tumor antigen NY-ESO-1 were shown to be effective at eliminating NY-ESO-1 positive cells in MM patients [43]. Therapeutic effects were limited in several patients by antigen escape and lack of T-cell persistence. CAR constructs provide T cells with the benefit of co-stimulatory signaling upon antigen engagement to promote persistence [2]. A CD19 targeting CART was shown to provide durable benefit in combination with ASCT in a patient with MM, despite the low prevalence of CD19 expressing cells [44]. Optimization of the adoptive cell therapy approach to accomplish durable clinical responses can be attempted through targeting of a highly prevalent antigen and selection of a robustly active CAR construct.

Tumor antigens that are targeted by monoclonal antibodies in the clinic are not necessarily suited to the CART approach due to the observed lack of discrimination between tumor and antigen positive, non-malignant cells [45]. The MM antigens CS1 and CD38 have reported expression on normal CD8+ T cells as well as other hematopoietic cell types, raising concerns about unwanted toxic effects on normal cells [46] as well as the potential for self-limiting activity due to CART fratricide [47]. BCMA is an attractive antigen for CART therapy due its limited expression on normal memory B-cell subsets and plasma cells and frequent expression in MM, where it contributes to tumor cell survival [23, 24]. We have verified the expression of BCMA on surface of MM cells from patients and expanded the assessment of the prevalence of this antigen in MM with our observation that, in our dataset, more than 90% of MM patients homogeneously express BCMA in clonal PCs.

We have described here the process undertaken to identify a robust clinical candidate BCMA-targeting CAR through the discovery and characterization of novel CAR constructs. Screening of a phage display library of scFv sequences derived from human B-cell antibody sequences was successful in identifying soluble scFv proteins that bind to BCMA antigen on the surface of cells. Engineering of CAR constructs containing these scFv sequences as the antigen recognition domain led to receptors that could be successfully expressed on the surface of T cells and maintain BCMA binding properties. *In vitro* activity assays revealed the functionality of these clones in T cells and discriminated clones with selective antigen recognition properties from non-selective clones. A xenografted myeloma cell mouse model confirmed anti-tumor activity *in vivo* and revealed clone 10 to be a potent clone. The scFv antigen recognition domain of clone 10 was found to specifically bind to BCMA expressing tissues and not antigen negative tissue.

Immunohistochemistry with commercially available polyclonal tool antibodies directed against human BCMA detected immunoreactivity in human multiple myeloma tissue and plasma cells. One antibody also unexpectedly produced weak to moderate staining in cerebellar climbing fibers, Brunner's glands of the duodenum and salivary gland. To further examine the source of immunoreactivity detected in non-lymphoid tissue with the polyclonal antibody, *in situ* hybridization was performed to detect BCMA mRNA. Using this sensitive technique, no mRNA was detected in salivary gland, Brunner's glands, cerebellum or inferior olivary nucleus. The absence of BCMA mRNA signal in cerebellum and inferior olivary nucleus was confirmed by RT PCR which failed to demonstrate BCMA mRNA in these structures. Moreover staining with a chimeric tool antibody incorporating the clone 10 scFv produced no immunoreactivity in the central nervous system. Based on these findings, the immunoreactivity detected in non-plasma cells by the rabbit polyclonal antibody is not consistent with BCMA protein expression within the CNS and very likely reflects cross reactivity of the commercial antibody with an unknown epitope(s). This conclusion is in agreement with previously published work using a goat anti-BCMA polyclonal antibody that failed to demonstrate BCMA expression on normal tissues outside of plasma cells (Carpenter *et al*, 2013).

While the likelihood of BCMA expression within the CNS was considered to be very low, none of the detection methods used have defined thresholds for positive results. We therefore cannot completely exclude the possibility that BCMA is expressed at low densities on cells within the CNS. Given the high sensitivity of CAR-T cells to very low densities of target antigen, we included the possibility of CNS expression of BCMA within the consent form for our phase I clinical trial, and we further incorporated rigorous assessment of neurologic function, especially fine motor control, by a neurologist performed at baseline and during post-treatment assessments out of an abundance of caution. Our experience using commercially available antibodies illustrates the critical importance of rigorous validation of antibodies used for preclinical assessments of CAR-T cell target antigens.

Carpenter, *et al*. [17] previously demonstrated that antibody Fab fragment sequences targeting BCMA could be converted into an scFv format binding element to constitute a functional CAR with anti-myeloma activity. The authors demonstrated *in vivo* activity of one clone up to 15 days and animal survival up to 30 days in a subcutaneous myeloma model. A key aspect of the work detailed here is the assessment of the anti-tumor activity of multiple clones *in vivo* in a prolonged study which revealed differences amongst the clones in their durability of anti-myeloma activity in a disseminated tumor model. Durability of clinical response in CD19 directed CART therapy has been correlated with the persistence of CART cells [33]. Therefore, durable and consistent *in vivo* activity of the BCMA-targeting CARTs when produced in T cells from 2 different donors was a critical parameter for selection of the clinical candidate construct. The consistency of durable activity of clone 10 in both studies suggests it may be more refractory than the other clones to donor variability. Based upon the selection of a highly active and specific BCMA-targeting CAR clone described here, we opened a clinical trial (NCT02546167) that is testing the safety and efficacy of autologous CART cells expressing BCMA CAR Clone 10 in patients with relapsed and/or refractory multiple myeloma. Early results from this Phase I clinical trial validate the preclinical work presented, demonstrating that the CAR derived from clone 10 is capable of mediating deep and durable responses in individuals with relapsed/refractory MM [21].

## MATERIALS AND METHODS

### Cell lines

K562 (DSMZ) cells were cultured in IMDM with 10% FBS. K562-BCMA cells were produced from K562 cells by lentiviral transduction of the BCMA gene under the control of the EF-1 alpha promoter along with Blasticidin selection marker. K562-BCMA cells were maintained in IMDM with 10% FBS and 2 ug/ml blasticidin. NCI-H929 and RPMI 8226 (ATCC) were cultured in RPMI with 10% FBS. KMS-11 cells were obtained under MTA from the Toronto General Research Institute and stable transfected with the luciferase gene to create the KMS-11-luc cell line. The identity of all cells lines was verified by SNP testing prior to use.

### Flow cytometry on MM samples

Mouse anti-human antibodies were purchased from multiple vendors (listed in Supplementary Table 1). Cells were washed once in PBS (Gibco, Life Technologies, 14190-136), supplemented with 2% fetal bovine serum (Gemini, 100-106, West Sacramento, CA) and stained for 15 minutes at room temperature. In all analyses, the population of interest was gated based on forward vs. side scatter characteristics followed by singlet gating, and live cells were gated using Live Dead Fixable Aqua (Invitrogen, LifeTechnologies, L34957) as shown in Supplementary Figure 1A. Time gating was included for quality control. Flow cytometry was performed on a four-laser Fortessa-LSR II cytometer or Accuri C6 (Becton-Dickinson, San Jose, CA) and analyzed with FlowJo X 10.0.7r2 (Tree Star, Ashland OR).

### ScFv library screening and CAR construction

M13 phage libraries displaying scFv's constructed from human B cells were used for panning and screening following a standard protocol [48]. Briefly, the libraries were first depleted with streptavidin beads and binding phages were captured by biotinylated human BCMA on magnetic beads. The panning process was repeated for 3 rounds and panning outputs from the final rounds were used to infect E. coli strain MC1061F'. Individual clones were subjected to sequencing. Periplasms from unique clones were prepared [49] and tested for binding to BCMA on cells. For cell surface binding assays, HEK293E cells were transiently transfected with a pLenti6.3 plasmid (Invitrogen) encoding the full length human BCMA cDNA downstream of the EF1alpha promotor following a standard lipofectamine 2000 protocol. Cells were analyzed by flow cytometry using Guava (easyCyte 8HT). Cell binding clones were then reformatted to express His-tagged ScFv proteins in E.coli culture supernatant (Miller *et al* 2010), from which scFv-His proteins were purified. The purified proteins were assessed for purity by SDS-PAGE. To retest cell binding, we mixed purified scFv-His proteins with anti-his-RPE (IC050P, R&D systems) and then added them to HEK293E cells with or without BCMA expression (as above). Cell binding was analyzed by flow cytometry.

For creation of CAR constructs, ScFv sequences were synthesized with a 5′ BamH1 site followed by leader and a 3′ silent BspE1 site within the hinge region sequence. The BamHI and BspEI sites were used to clone into the pELPS lentiviral vector as in [50].

### CART cell production and characterization

Replication defective lentivirus was produced by standard methods using the CAR plasmid mixed with the three packaging components of VSVg, gag/pol and rev, and transfected into LTX-293T cells (Clontech) using Lipofectamine 2000. Lentiviral titer was determined by transduction of Sup-T1 cells and assessment of cell surface CAR expression as described below. For CART cell preparation, isolated T cells were derived from blood obtained with consent through the Novartis Employee Blood Donor program. T cells were isolated from Ficoll separated PBMCs by negative selection using the Pan T Cell Isolation Kit II (Miltenyi Biotec). T cells were expanded as in [50]. Lentiviral supernatants were added 24 hours after bead addition at MOI = 5. No virus was added to untransduced T cells. T cells were split every 2 to 3 days to a concentration of 0.5 to 0.8 million cells per ml. 10 days after expansion, T cells were magnetically de-beaded and harvested for further analyses.

CAR expression was determined on transduced SupT1, JNL, or primary T cells as follows. Transduced cells were pelleted, washed once with MACS buffer containing BSA (FACS buffer), and resuspended in FACS buffer at 1 × 10^6^ cells/ml. Recombinant human BCMA-Fc protein (Novartis) was added at 1 ug/ml concentration and incubated at 4°C for 30 minutes. Cells were washed twice with FACS buffer and stained with Alexa Fluor 647-conjugated anti-human secondary antibody (Jackson Immuno Research) for 15 minutes. Cells were washed twice and resuspended in FACS buffer. Samples were measured on a flow cytometer, and data were analyzed with FlowJo software.

### JNL activity assay

The JNL reporter cell line was engineered from Jurkat E-6 cells and contains the firefly luciferase gene under the control of a minimal (m) CMV promoter and tandem repeats of the NFAT transcriptional response element. Cells were cultured in RPMI with 10% FBS and 0.5 μg/ml puromycin. JNL cells transduced with BCMA-targeting CAR constructs were evaluated for activation in response to BCMA-expressing target cell lines. On Day 3 following transduction, transduced JNL cells were incubated with target cells in duplicate at an effector-to-target (E:T) ratio of 6:1. JNL activation was measured using Bright-Glo substrate (Promega) on Day 4.

### CART functional assays

CART cell proliferation in response to BCMA-expressing target cells was evaluated as in [50] by co-culturing transduced or untransduced T cells with irradiated target cell lines (K562, K562-BCMA, NCI-H929, KMS-11-luc, and RPMI 8226) for 4 days. CART cell killing was performed by co-culturing CART cells with KMS-11-luciferase target cells at different E:T ratios for 20 hours as in [50]. CAR T cell populations were normalized to equivalent percentages of CAR+ cells before plating. Cells were stained with anti-CD3 antibody (PerCPCy5.5, eBioscience) and measured by flow cytometry relative to CountBright Absolute Counting Beads (Life Technologies) to determine relative cell counts. Cytokines IL-2 and IFNγ were measured in supernatants from 20 hour co-cultures of CART cells with target cells in duplicate at effector to target ratio of 2.5:1 using the Meso Scale Discovery (MSD; Gaithersburg, MD) Proinflammatory Panel 1 (human) Kit assay platform as per the manufacturer's protocol. Plates were read on the MESO sector S 600, and the results for each cytokine were calculated in pg/ml using known standards. All assays were performed in duplicate from a single source of donor cells. Assays with Clones 4, 9, 10, 13, and 15 have been repeated with cells derived from a second donor with similar results (data not shown).

### *In vivo* tumor models

All mouse experiments were performed according to Institutional Animal Care and Use Committee (IACUC)-approved protocols. Female NOD.Cg-*Prkdc^scid^Il2rg^tm1Wjl^/*SzJ (NSG) mice were implanted with 1 × 10^6^ KMS-11-luc tumor cells via the lateral tail vein. 7 days after tumor implantation CART cells were dosed via tail vein in 100 μl volume per animal. CAR T cell populations were normalized to equivalent percentages of CAR+ cells with untransduced T cells prior to injection such that the total number of T cells injected per mouse was equivalent across groups. Tumor burden was monitored twice weekly by bioluminescence imaging. For tumor measurements, mice were weighed and injected intraperitoneally with a 150 mg/kg dose of a firefly D-luciferin sodium salt monohydrate solution (Biosynth). Ten minutes following the luciferin injection, mice were anesthetized and imaged on the Xenogen IVIS-200 (Perkin Elmer). Using the Living Image software (Perkin Elmer), tumor measurements were calculated as the bioluminescence intensity of the entire animal in photons/second. Changes in body weight were monitored during the course of the study.

Antibodies used for flow cytometry of samples from mouse xenograft studies were anti-mouse CD11b Alexa Fluor 700 (BD Biosciences, catalog# 557960), anti-human CD45 BUV395 (BD Biosciences, catalog# 563795), anti-human CD4 PE-Cy7 (BD Biosciences, catalog# 557852), anti-human CD8 PerCP-Cy5.5 (BD Biosciences, catalog# 560662), BCMA-Fc for CAR detection (Novartis), and anti-human IgG Fcγ F(ab')2 (Jackson ImmuoResearch, catalog# 109-116-098).

The presence and expansion of total T cells and CAR T cells in peripheral blood from xenografted mice were analyzed via flow cytometry using antibodies itemized above. After processing (below), samples were run on a BD LSR Fortessa FACS machine (BD Biosciences). Data were analyzed using FlowJo analysis software (TreeStar, Inc.).

Peripheral blood was collected via tail snip (IACUC# 13-ONC-006; SOP# 524-061-01 Blood Collection in Mice). Mice were manually restrained and bled via the tail vein into EDTA-coated blood collection tubes. Blood was kept on ice and then immediately plated into 96 well plates for red blood cell lysis and staining for flow cytometry as described below. After the mice were bled via the tail, 8–10 μl of whole blood was plated per mouse per well in a 96 well plate on ice. Blood samples were then lysed with ACK lysing buffer (Gibco, catalog# A10492-01) for 1–2 minutes. The lysis was stopped by the addition of PBS to each well, and the samples were spun for 5 minutes at 1200 rpm. Following this, the samples were blocked with a 1:50 dilution of a mouse and human Fc block (Miltenyi Biotec, mouse – catalog# 130-092-575; human – catalog# 130-059-091) mixture for 20 minutes on ice. The samples were spun again for 5 minutes at 1200 rpm and then stained with a 1:200–1:400 dilution of each primary antibody for 30 minutes on ice in the dark. The plate was spun again for 5 minutes and then stained with a 1:1000 dilution of the Fcγ secondary antibody for 20–30 minutes on ice in the dark. Following this, the plate was spun again for 5 minutes at 1200 rpm and then fixed with 2% paraformaldehyde (PFA) for 20 minutes on ice in the dark and then stored in fluorescence activated cell sorting (FACS) buffer (PBS with 2% fetal bovine serum) for subsequent analysis.

### Affinity measurement

Recombinant human BCMA-Fc was obtained from R&D Systems and chemically biotinylated with NHS-Peo-Biotin from Thermo-Fisher Scientific according to the manufacturer's instructions. Biointinylated rhBCMA-Fc (Novartis) was immobilized to a streptavidin coated sensor chip at a density of 150 RU. The plasmid encoding the amino acids for the scFv construct was synthesized externally. The scFv was produced transiently in HEK293F cells and purified using the 8xHis tag on the C-terminus of the constructs with standard methodology. Briefly, 100 ml of HEK293F cells at 3 × 10^6^ cells/ml were transfected with 100 μg plasmid and 300 μg polyethylenimine. The cells were incubated at 37°C with 8% CO_2_ and rotated at 80 rpm. After six days, the cells were harvested by centrifugation at 3500 g for 20 minutes. The supernatant was purified by binding the scFv to 200 μl Ni-NTA agarose beads (Qiagen) overnight at 4°C. The protein was eluted with 200 μl 300 mM imidazole, and dialyzed against phosphate buffered saline. The scFv sample was serially diluted 3-fold and injected over the chip at a constant flow rate. Association and dissociation rates of the protein complex were monitored for 270 s and 400 s, respectively. Double referencing was performed against a blank immobilized flow cell and a buffer blank and the data was fit using a 1:1 Langmuir model with the Biacore T200 evaluation software.

### Immunohistochemistry and *in situ* hybridization

Immunohistochemistry was performed using polyclonal antibodies B0807-50G (US Biological) and AF193 (R&D systems). In brief, FFPE tissues were sectioned at 5μm, bar coded and then placed in the autostainer. Antigen retrieval, primary antibody dilution, incubation temperature and duration, detection technique and chromogen were optimized on non-study archived tissues and included evaluation of an irrelevant antibody control and known negative and positive tissues and cell lines where appropriate. Staining was completed on a Ventana Ultra Discovery using 60 min primary incubation at a concentration of 0.6 μg/ml for B0807-50G and 5.0 μg/ml for AF193. A human tissue microarray was stained and evaluated using both the BCMA10 chimeric antibody as well as an isotype matched irrelevant chimeric antibody control. Normal human tissues were graded (0 to 5) for BCMA staining intensity with a score of 0 indicating an absence of staining and a grade of 5 indicating intense uniform staining.

*In situ* hybridization was performed using Advanced Cell Diagnostics (ACD)/Ventana systems probes and reagents. The BCMA probe (cat#585797) was designed by ACD using accession number NM_001192.2 covering the region of nucleotides 31-984. The reagents utilized: 1) RNAscopeVS probe sets including positive control probe PPIB (Cat#313906-C2) and DapB negative control probe (Cat#310048), 2) RNAscopeVS FFPE reagent kit (Cat#320600) including Pretreat A, pretreat B, and Amp1 through 7, 3) RNAscopeVS FFPE accessory kit (Cat#320630) and 4) RNAscopeVS FFPE offline CC Kit (Cat#320043) including 10x pretreat 2 solution. For the Ventana automated system the reagents used were 1) mRNA DAB detection kit (Cat#760-224), 2) mRNA probe amplification kit (Cat#760-222), 3) mRNA pretreatment kit (Cat# 760-223) and 4) Probe dispensers for ACD probes (Cat# 960-76X such as probe1 cat#960-761 and probe2 cat#960-762). The ISH method followed protocols established by ACD Bio and Ventana systems. Briefly the sections were baked at 60 degrees for 30 minutes. The protocol was 3 steps xylene for 3 minutes; 2 times 100% alcohol for 3 minutes; once 95% alcohol for 3 minutes; followed by one step 80% alcohol for 3 minutes; distilled water rinse for one minute; and tap water for 2 min. Rehydration was follow by offline tissue conditioning at 99 degree for 15 minutes. Finally, the slides were transferred to Ventana Ultra for finishing the ISH procedure including protease pretreatment; hybridization and amplification for three hours; and detection with HRP and hematoxylin counter stain.

To produce a soluble recombinant antibody for immunohistochemistry containing the ScFv sequences of CAR Clone 10 fused to a rabbit Fc region, the sequences for the variable light and heavy chain coding regions were cloned into a mammalian expression vector. This plasmid was transiently transfected into HEK293 Freestyle cells and the antibody was purified from the supernatant and confirmed by mass spec.

For immunohistochemistry, formalin fixed paraffin embedded tissues were sectioned at 5μm and stained in a Ventana Ultra autostainer The BCMA 10 rabbit human chimeric antibody was used at 0.35 μgm/ml and immunohistochemistry performed as described above.

### BCMA qPCR

For BCMA qPCR synthesis of cDNA was carried out using 500 ng of total RNA per sample using the High-Capacity cDNA Reverse Transcription Kit (Applied Biosystems, catalog # 4368814) per the manufacturer's instructions. qPCR reactions were performed using the TaqMan Gene Expression Master Mix (Applied Biosystems, catalog #4369016) per the manufacturer's instructions, using a 40 μL reaction volume and 25 ng cDNA per reaction. Real-time PCR amplification of cDNA samples was carried out on the ABI 7900HT Real-Time PCR instrument (Applied Biosystems) using commercially available TaqMan PCR probes. Commercially available TaqMan probes were used for cDNA amplification (Eukaryotic 18s4352930E, Monkey BCMA (TNFRSF17) Rh02837830_m1 and Human BCMA (TNFRSF17) Hs03045080_m1). RT-qPCR was performed in triplicate, and raw data was analyzed by tissue type, utilizing a relative quantification method assuming equivalent reaction efficiencies of the reference gene (18S ribosomal RNA) and BCMA. After analysis, ratios of BCMA expression over 18S ribosomal RNA expression were graphed in arbitrary units.

## SUPPLEMENTARY MATERIALS FIGURES AND TABLE


