# Supplementary material for: Pre-clinical validation of B cell maturation antigen (BCMA) as a target for T cell immunotherapy of multiple myeloma
Source: Oncotarget. 2018 May 25;9(40):25764–80. doi: 10.18632/oncotarget.25359 (PMC5995247; doi:10.18632/oncotarget.25359)
Supplement: Supplementary file 1 [file oncotarget-09-25764-s001.pdf]

Pre-clinical validation of B cell maturation antigen (BCMA) as a target for T cell immunotherapy of multiple myeloma

SUPPLEMENTARY MATERIALS

Supplementary Table 1: Demographic Characteristics of Subjects Analyzed in Figure 1B.

Subject#	Sample Type	Gender	Age	Diagnosis
1	BM			Multiple myeloma
2	PB	F	61	Plasma cell leukemia
3	BM	M	60	Multiple myeloma
4	BM	M	72	Plasma cell leukemia
5	PB	F	53	Plasma cell leukemia
6	BM	M	69	Multiple myeloma
7	BM	M	63	Multiple myeloma
8	BM	F	56	Multiple myeloma
9	BM	F	59	Multiple myeloma
10	BM			Multiple myeloma

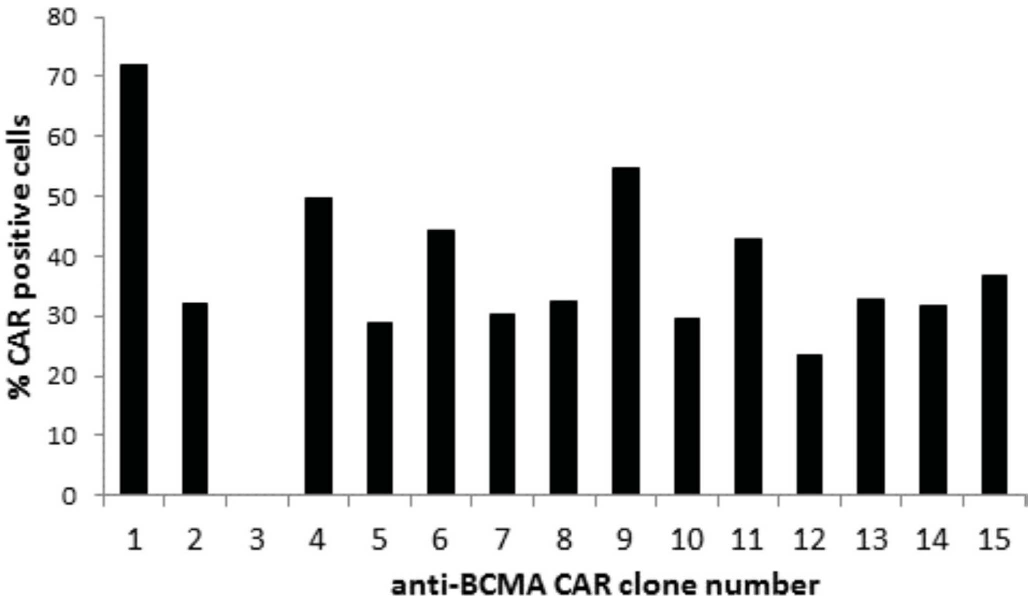

Supplementary Figure 1: Evaluation of the expression CAR constructs with antigen binding domains derived from the novel scFv sequences. The expression of the CAR clones on the surface of transduced T cells was evaluated by flow cytometry using recombinant BCMA as a detection reagent. Quantitation of CAR expression on the surface of primary T cells (% CAR+) is shown for each of the different CAR clones.

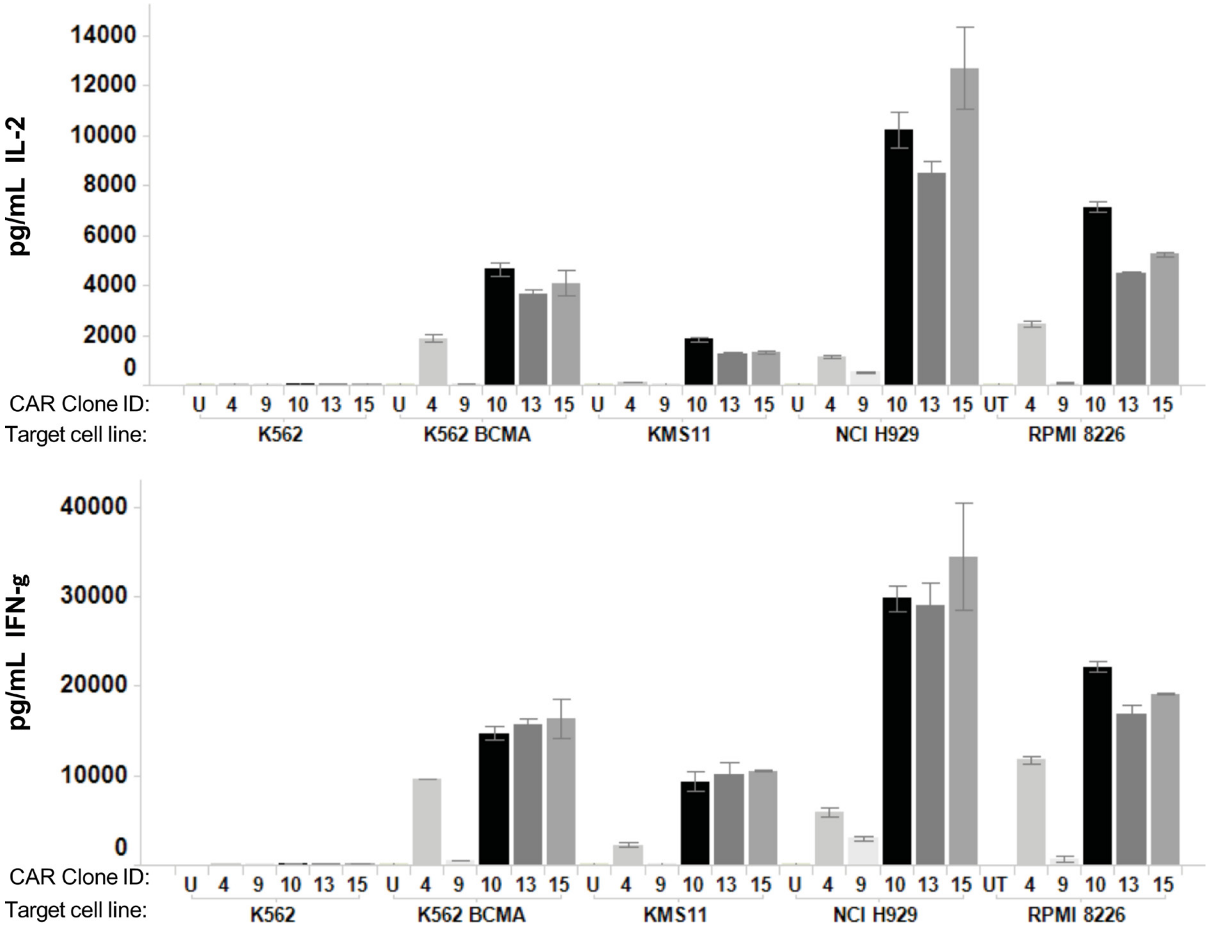

Supplementary Figure 2: BCMA-specific cytokine production by individual CART-BCMA clones. T cells expressing the indicated CAR constructs were evaluated for their ability to produce cytokines when co-cultured with antigen positive (K562-BCMA, KMS11-luc, NCI-H929, RPMI8226) and not antigen negative (K562) cell lines at an effector to target ratio of 2.5:1. Cytokines interleukin-2 (IL-2) and interferon-gamma (IFN-γ) were measure by MSD from supernatants collected from co-cultures of T cells with target cells. All data is expressed as the average ± standard deviation.

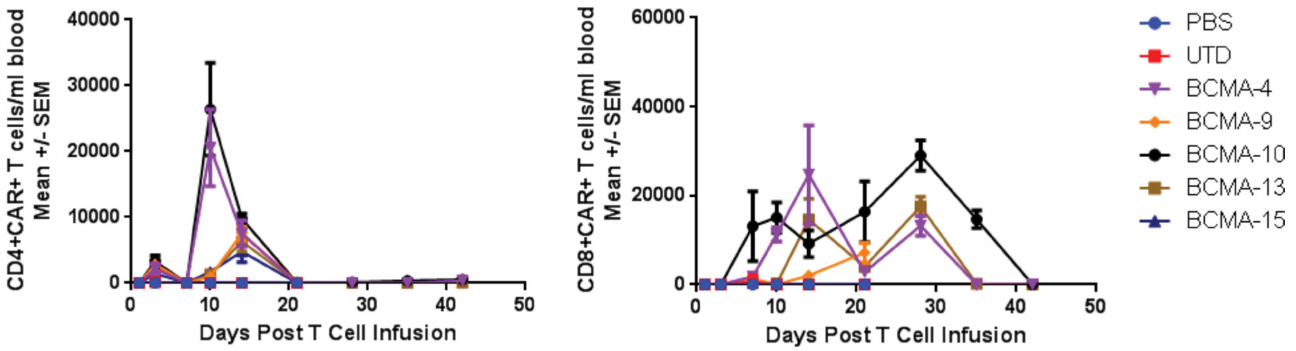

Supplementary Figure 3: T cell engraftment in KMS11-luc in vivo model following CART-BCMA treatment. The expansion of CART cells in the mice described in Figure 4E was determined by monitoring the presence of CAR+ cells in peripheral blood by flow cytometry. Cells were stained with anti-human CD45 and anti-murine CD11b to define human cells followed by anti-CD4 and anti-CD8 antibodies as well as with recombinant BCMA protein to detect CAR+ T cells. The number of CD4+ CAR$^+$ T cells (left panel) and CD8+ CAR+ T cells (right panel) detected in blood over time is shown.

A

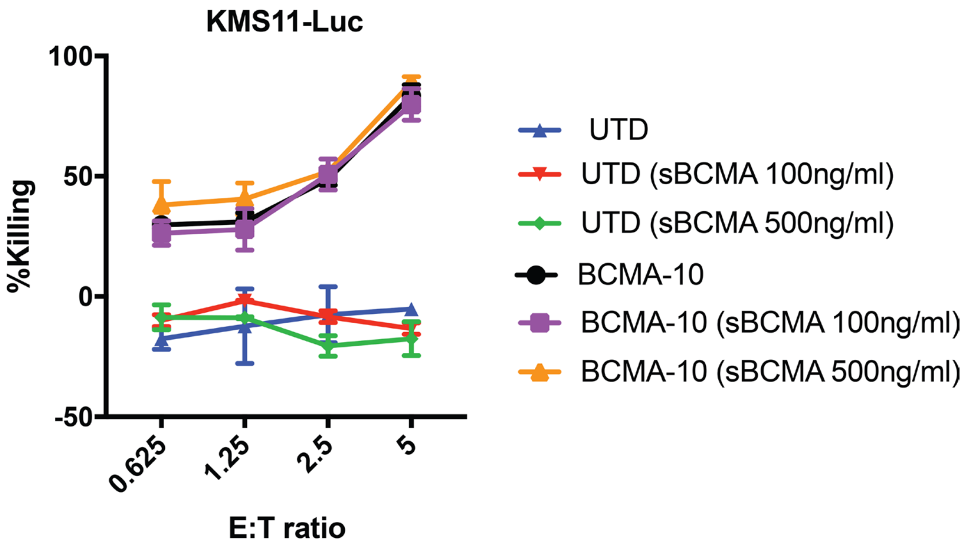

B

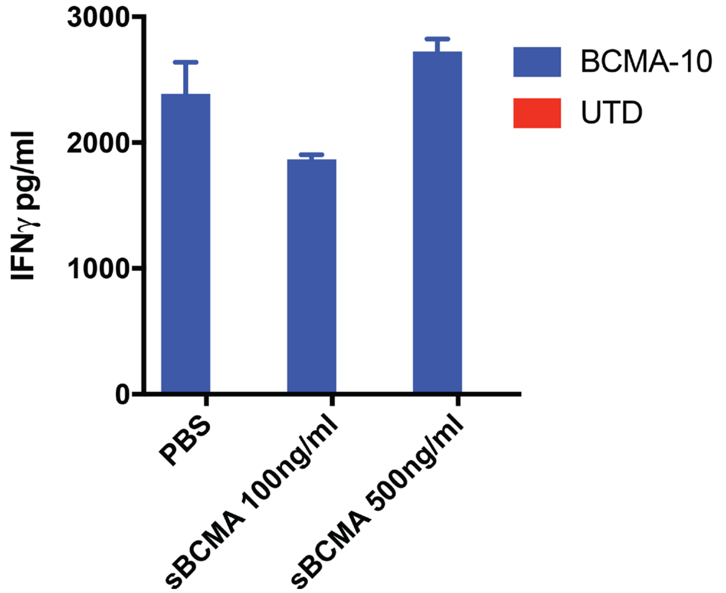

Supplementary Figure 4: Clone 10 CAR-stimulated cytotoxicity and cytokine production are unaffected by up to 500 ng/mL of sBCMA. (**A**) Specific cytotoxicity was measured as in Figure 4D by monitoring the level of luciferase produced by 8×10^3 tumor target KMS11-Luc (T) cells at 16 h after incubation with effector UTD or BCMA-10 CART cells (E) at different E:T ratios. The mixed cell culture was either treated with PBS or sBCMA (BCMA-ECD [aa1-54], Novartis) at 100 or 500 ng/ml. (**B**) IFNγ was measured by the Meso Scale Discovery (MSD; Gaithersburg, MD) Proinflammatory Panel 1 (human) Kit in these supernatants collected at 16 h from the co-culture system. Data are expressed as the mean ± SD of triplicate samples from 1 of 2 donors, with similar results.

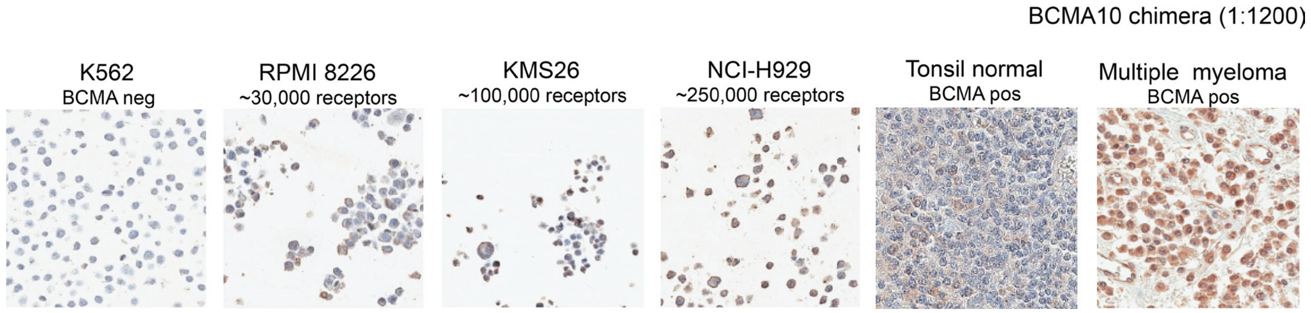

Supplementary Figure 5: Detection of BCMA at varying surface densities using clone 10 scFV expressed as a full length chimeric antibody. The indicated cell lines were cytospun onto glass slides and fixed in formaldehyde prior to IHC staining a chimeric antibody containing the clone 10 scFv. Normal tonsil and bone marrow with multiple myeloma were used as negative and positive controls, respectively. Staining shown was performed using 1:1200 dilution of the chimeric antibody, and an HRPconjugated, anti-rabbit secondary antibody.
